# Utility of transepidermal water loss‐stratum corneum hydration ratio in atopic dermatitis

**DOI:** 10.1111/srt.13709

**Published:** 2024-05-20

**Authors:** Shreya Sreekantaswamy, Jason Meyer, Erin Grinich, Yael Leshem, Eric Simpson, Katrina Abuabara

**Affiliations:** ^1^ San Francisco Department of Dermatology University of California San Francisco USA; ^2^ Department of Dermatology University of Utah Salt Lake City USA; ^3^ Department of Dermatology Vanderbilt University Medical Center Nashville USA; ^4^ Department of Dermatology Oregon Health & Science University Portland USA; ^5^ Division of Dermatology Rabin Medical Center Petah Tikva Israel; ^6^ School of Medicine Tel Aviv University Tel Aviv Israel; ^7^ Division of Epidemiology and Biostatistics University of California Berkeley USA

**Keywords:** atopic dermatitis, SCH, stratum corneum hydration, TEWL, transepidermal water loss

1

The epidermis plays a key role in maintaining homeostasis via its regulation of water loss through the skin. Epidermal water barrier and binding characteristics are typically assessed via transepidermal water loss (TEWL) and stratum corneum hydration (SCH), respectively. Given that TEWL is a measure of water vapor transport out of the skin, one would expect that TEWL would increase in conditions with barrier dysfunction; however, not only has research shown that TEWL does not always increase in such conditions,^1^ but TEWL has also been found to have high variability in normal skin.^2^

One proposed explanation for this phenomenon is that the epidermis does not detect or regulate TEWL directly, but rather responds to changes in water content in the stratum granulosum (SG). In moist conditions (such as high humidity, sweating, or skin occlusion), hydration in the SC (SCH) and SG increase. Since in these conditions the epidermis does not require a strong barrier to maintain water in the SG, the barrier is allowed to remain permeable.^3^ Conversely, in dry conditions (low humidity, xerosis) SCH and SG hydration fall, so a less permeable barrier is required.^3^ It can therefore be postulated that there is a correlation between SCH and TEWL. When a compensatory response, triggered by a deviation in SG hydration from homeostasis, is unsuccessful in normalizing SG hydration, an altered ratio of TEWL:SCH may persist. Such a deviation in TEWL and SCH has been demonstrated in psoriasis^4^ and workers exposed to low humidity conditions.^5^ We hypothesized that a TEWL:SCH ratio could detect deviations in barrier homeostasis in atopic dermatitis (AD), potentially serving as a more discriminatory measure in ascertaining subtle differences after treatment or in identifying changes in some subpopulations than either TEWL or SCH alone.

We identified two previously published studies which had recorded both TEWL and SCH measurements in the volar forearms of participants aged 17 and older with and without AD. The first, by Grinich et al.,^6^ tested the validity of measurements of epidermal barrier function by the GPower GPSkin device compared to the gold standard devices for TEWL (Aquaflux) and SCH (Corneometer) on 50 healthy controls and 50 patients with AD (on both lesional and nonlesional skin). The second study, by Leshem et al.,^7^ included 8 healthy controls and assessed skin barrier function pre and post emollient treatment in 25 patients with AD, with one arm serving as a control and one arm receiving emollient. Data from Aquaflux and Corneometer devices in both studies were utilized for our analyses.

We compared the performance of the TEWL:SCH ratio to TEWL and SCH alone to (1) differentiate nonlesional skin in AD from healthy controls, (2) differentiate disease severity in nonlesional AD skin, and (3) identify changes after emollient treatment in nonlesional AD skin (Figure 1). For each comparison, we calculated the area under the ROC curve,^8^ as shown in Table 1. TEWL alone best differentiated nonlesional skin in AD from healthy controls (aROC 0.68) and disease severity (aROC 0.84). Variance was high in the SCH of AD lesional skin, consistent with heterogeneity in the clinical presentation of AD. For example, acute lesions have been described as exudative while chronic lesions may be more likely to be xerotic and lichenified. The TEWL:SCH ratio performed slightly better in differentiating changes in the treatment arm from the control arm after emollient use (aROC 0.63) and in differentiating AD severity in the treatment arm after emollient use (aROC 0.80).

**FIGURE 1:**
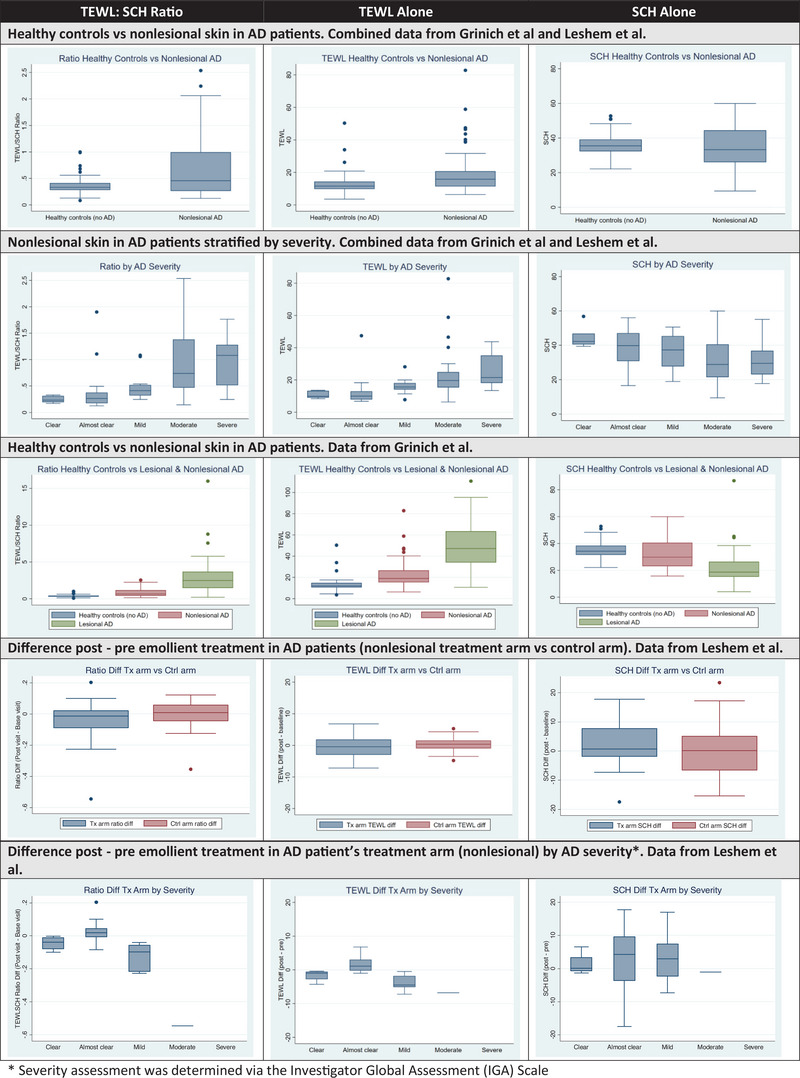
Distribution of the TEWL:SCH ratio versus TEWL alone versus SCH alone in data from Grinich et al. and Leshem et al. SCH, stratum corneum hydration; TEWL, transepidermal water loss.

**TABLE 1:** Summary of data from Grinich et al. and Leshem et al.

Combined data from Grinich et al. and Leshem et al.										
		TEWL:SCH Ratio			TEWL			SCH		
	N	Mean	SD	aROC	Mean	SD	aROC	Mean	SD	aROC
Controls	58	0.37	0.17	0.63	13.10	6.87	0.68	36.03	6.71	0.54
AD patients^a^	75	0.68	0.57		19.14	12.92		34.87	11.67	
Clear	6	0.24	0.07	0.82^b^	10.69	2.23	0.84^b^	44.65	6.53	0.72^b^
Almost clear	19	0.39	0.42		12.37	9.08		39.14	10.77	
Mild	12	0.50	0.28		16.28	4.97		36.57	10.29	
Moderate	26	0.94	0.67		24.24	16.51		30.54	12.25	
Severe	12	0.96	0.49		25.87	10.17		30.93	10.62	

Comparing lesional versus nonlesional skin in AD patients. Data from Grinich et al.										
Lesional skin	48	2.96	2.61	0.87	23.15	22.76	0.85	22.70	13.23	0.76
Nonlesional skin	50	0.85	0.85		49.24	14.02		31.84	10.99	

Post—Pre treatment data from Leshem et al.										
		TEWL:SCH Ratio Difference			TEWL Difference			SCH Difference		
	N	Mean	SD	aROC	Mean	SD	aROC	Mean	SD	aROC
Control arm	24	−.00	0.10	0.63	0.30	3.64	0.59	−0.35	9.14	0.62
Treatment arm	24	−0.04	0.14		−0.49	2.36		2.57	8.01	
Clear	4	−0.05	0.04	0.80^c^	−1.68	1.79	0.78^c^	1.3	3.54	0.59^c^
Almost clear	12	0.03	0.07		1.67	2.32		2.82	9.70	
Mild	6	−0.12	0.08		−3.93	2.42		3.43	8.68	
Moderate	1	−0.55			−6.8			−1.1		
Severe	0									

Abbreviations: AD, Atopic dermatitis; SCH, stratum corneum hydration; TEWL, transepidermal water loss.

^a^ Measurements taken from nonlesional skin in AD patients (pr‐treatment values from Leshem et al.).

^b^ Comparing clear—mild versus moderate—severe.

^c^ Comparing clear—almost clear versus mild—moderate.

Our study was limited by a lack of data on participant ages.^6^, ^7^ Skin barrier function declines with age due to changes in lipid processing and acidification; however, most studies have found that TEWL paradoxically improves (decreases) with age.^9^, ^10^ Future studies should therefore also examine whether TEWL:SCH helps to differentiate barrier function specifically in the older adult population.

In summary, we found that the overall difference between the TEWL:SCH ratio and TEWL alone was negligible, however, TEWL alone best distinguished AD from controls and AD severity status (on non‐lesional skin), whereas the TEWL:SCH ratio may have been more sensitive to changes after emollient use. Given that dermatology trials are limited by a lack of quantitative objective outcome measures, even small improvements in discriminatory capacity are important. The TEWL:SCH ratio therefore warrants further testing and may be of use to researchers designing future trials.

## CONFLICT OF INTEREST STATEMENT

Yael Leshem has received honoraria or fees for consultation/Speakers Bureau participation from AbbVie, Sanofi, Janssen, Pfizer, Eli‐Lilly and Genentech, and as an advisory board member from Sanofi, Regeneron, Pfizer, Eli‐Lilly, AbbVie, and Dexcel Pharma; has received an independent research grant from AbbVie; and has, without personal compensation, provided investigator services for Eli Lilly, Pfizer, Sanofi, and AbbVie. Katrina Abuabara has received honoraria or fees for consultation/ Speakers Bureau participation from Sanofi, Nektar, Amgen, and TARGET RWE, and has received independent research grants to her institution from Pfizer and Cosmetique Active International SNC. Dr. Eric Simpson reports personal fees from AbbVie, Amgen, Arcutis, Areteia Therapeutics, Bristol Myers Squibb BMS, CorEvitas, Corvus, Dermira, Eli Lilly, Evelo Biosciences, FIDE, Forte Bio RX, Galderma, GlaxoSmithKline, Gilead Sciences, Impetus Healthcare, Incyte, Innovaderm Reche, Janssen, Johnson & Johnson, Kyowa Kirin Pharmaceutical Development, Leo, Merck, MJH holding (4/29/2021), NUMAB Therapeutics AG, Pfizer, Physicians World LLC, PRImE, Recludix Pharma, Regeneron, Roivant, Sanofi‐Genzyme, SITRYX Therapeutics, Trevi therapeutics, Valeant. Dr. Eric Simpson reports grants (or serves as Principal investigator role) for AbbVie, Acrotech, Amgen, Arcutis, ASLAN, Castle, CorEvitas, Dermavant, Dermira, Incyte, Lilly, Kymab, Kyowa Kirin, National Jewish Health, Leo, Pfizer, Regeneron, Sanofi, Target, VeriSkin.

## Data Availability

Data sharing is not applicable to this article as no new data were created or analyzed in this study.
